# Monitoring *EGFR*-T790M mutation in serum/plasma for prediction of response to third-generation EGFR inhibitors in patients with lung cancer

**DOI:** 10.18632/oncotarget.25478

**Published:** 2018-06-05

**Authors:** Teresa Morán, Eudald Felip, Joaquim Bosch-Barrera, Itziar de Aguirre, Jose Luis Ramirez, Carles Mesia, Enric Carcereny, Diana Roa, Elia Sais, Yolanda García, Remei Blanco, Silvia Sanchez, Claudia Rosa Villacorta, Cristina Queralt, Jose María Velarde, Rafael Rosell

**Affiliations:** ^1^ Medical Oncology Department, Catalan Institute of Oncology - Badalona, Hospital Universitari Germans Trias i Pujol, Badalona, Universitat Autònoma de Barcelona (UAB), Barcelona, Spain; ^2^ Medical Oncology Department, Catalan Institute of Oncology-Girona, Hospital Universitari Doctor Trueta Girona, Girona, Spain; ^3^ Molecular Biology Laboratory of Cancer Dr. Rosell, Can Ruti Campus: Institute Germans Trias i Pujol (IGTP), Catalan Institute of Oncology, Badalona, Spain; ^4^ Medical Oncology Department, Catalan Institute of Oncology - Hospital Duran i Reynalds, l’Hospitalet de Llobregat, Barcelona, Spain; ^5^ Medical Oncology Department, Hospital Parc Taulí, Sabadell, Barcelona, Spain; ^6^ Medical Oncology Department, Consorci Sanitari de Terrassa, Terrassa, Barcelona, Spain; ^7^ Research Nurse Team, Catalan Institute of Oncology - Badalona, Hospital Universitari Germans Trias i Pujol, Badalona, Spain; ^8^ Statistics Department, Fundació Germans Trias i Pujol, Badalona, Spain

**Keywords:** EGFR-T790M mutation, serum/plasma, osimertinib, acquired resistance, EGFR tyrosine kinase inhibitors

## Abstract

**Background:**

Osimertinib is efficacious in lung cancer patients with epidermal growth factor receptor (*EGFR*) mutations and acquired resistance (AR) to EGFR tyrosine kinase inhibitors due to *EGFR*-T790M mutation (T790M). We sought to describe T790M changes in serum/plasma during osimertinib therapy and correlate these changes with treatment outcomes.

**Material and methods:**

Serum/plasma from *EGFR*-mutant lung cancer patients with T790M-AR was collected before and during osimertinib treatment. Changes in T790M were evaluated using a peptide-nucleic acid-PCR assay, and correlated with clinical and radiographic response.

**Results:**

Thirteen patients were included. Median time on osimertinib treatment was 10.6 months with a median progression-free survival of 13.6 months. Best response to osimertinib was partial response (PR), stable disease (SD) or progression (PD) in 46.1%, 30.8% and 23.1% of patients, respectively.

Most of the patients were paucisymptomatic at baseline. Symptom improvement was reported in 66.6% of responder patients; while symptoms remained stable in 75% of patients with SD, and 66% of patients with PD had clinical deterioration.

Three patterns of T790M changes during osimertinib treatment were identified. T790 remained detectable with PD or a short-lasting SD in 15.4% of the patients. T790M disappeared in 69.2% of patients with PR or SD. T790M disappeared, despite clinical and/or radiographic progression in 15.4% of the patients.

**Conclusion:**

Changes of T790M in serum/plasma in *EGFR*-mutant lung cancer patients with T790M-AR might be a useful marker of symptomatic and radiographic outcome to osimertinib. Longer follow-up is needed to establish if subsequent emergence of T790M could be a marker of resistance.

## INTRODUCTION

First- and second-generation tyrosine kinase inhibitors (TKIs), erlotinib, gefitinib and afatinib, as well as the combination of erlotinib and bevacizumab, have demonstrated a clinically meaningful benefit as upfront therapy in patients harboring epidermal growth factor receptor (*EGFR*) mutations [1]. However, despite an initial clinical benefit, acquired resistance (AR) invariably develops, mainly due to the emergence of the secondary mutation *EGFR*-T790M (T790M) [2, 3]. Several clinical trials have tested different drugs to treat patients with AR to first- and second-generation TKIs with modest results [4–10]. Therefore, standard chemotherapy and best supportive care have remained the main treatment options for these patients. Currently, third-generation TKIs with the ability to inhibit both T790M and the sensitizing *EGFR* mutations have entered the clinical scenario [11, 12]. Osimertinib, a third-generation TKI, has demonstrated a clinically meaningful benefit in patients with *EGFR* mutations who have developed AR to TKIs. In clinical studies with osimertinib, patients with T790M-positive tumors reported an overall response rate (ORR) of 61% and median progression-free survival (mPFS) of 9.6 months, with a favorable toxicity profile [12]. Recently, osimertinib has also demonstrated significant advantages in terms of response and progression-free survival in *EGFR*-mutant lung cancer patients treated in first line [13].

One limitation for *EGFR* mutation testing is the scarcity of tumor tissue in patients with advanced disease. Detecting *EGFR* mutations in serum or plasma has been proposed as an alternative to tumor tissue. Using serum and plasma for molecular testing relies on the fact that mutant DNA fragments from necrotic neoplastic cells are freely circulating in blood [14]. As tumors become more aggressive there is more necrosis, leading to an increase in the absolute amount of circulating DNA that can be detected. Initial studies report that *EGFR* mutations could be detected in paired tumor and plasma samples in more than 70% of the patients with *EGFR*-mutant lung cancer [15]. Moreover, different sources of tumor DNA present in the peripheral blood provide dynamic information of the disease, reflecting not only the assessment of the primary tumor, but also of the metastatic sites.

Different techniques, including emulsion polymerase chain reaction (PCR) (ddPCR and ddPCR Beaming), reverse transcription PCR (RT-PCR), peptide-nucleic acid (PNA), amplification refractory mutation system (ARMS), and Scorpion, have been used to detect *EGFR* mutations with a sensitivity ranging from 61% to 93% for the sensitizing mutations and from 41% to 81% for T790M [16]. *EGFR* mutations in serum and plasma have been used to monitor the evolution of the sensitizing mutations during treatment. In addition, the presence of *EGFR* mutations in serum and plasma has been associated with poorer prognosis [17, 18]. Recently, peripheral blood has become a useful source to detect the presence of T790M as a mechanism of AR [19]. Interestingly, T790M can be detected in 70% of the patients with AR to first- and second-generation TKIs, which implies that tumor rebiopsy could be obviated in patients with positive result in plasma. ORR and mPFS were identical in both T790M– plasma-positive and tumor-positive patients. In addition, urine samples were also evaluated concurrently with serum and tissue samples in patients who received treatment with rociletinib, another third-generation EGFR-TKI [20]. Together, urine and plasma samples identified a higher proportion of T790M than tissue alone (89% vs. 75%), and T790M levels decreased in the urine samples of patients who responded or had stable disease (SD) shortly after starting treatment.

For the present study, we postulated that if detected at baseline in serum/plasma once AR to first- and second-generation TKIs has occurred, T790M mutation could be monitored during treatment with a T790M TKI. We hypothesized that this T790M monitoring in *EGFR* mutant patients with a T790M mutation as an AR mechanism receiving a T790M TKI could indicate response to therapy and subsequent progressive disease (PD). Moreover, T790M loss during treatment could potentially be correlated to clinical and radiographic response and to a quick time to response, should T790M disappear in plasma/serum. Thus, the follow-up of T790M mutation in plasma/serum could be used as a monitoring tool of response in patients receiving a T790M inhibitor.

## RESULTS

Twenty-one patients with *EGFR* mutations were treated with osimertinib between January 2016 and June 2017 after confirmed PD to a prior TKI. All the patients harbored the T790M mutation, which was evaluated in serum/plasma and also in tissue when available. Only patients with T790M in serum/plasma were eligible. Eight patients were excluded from the analyses because T790M was only detected in tissue, but not in serum/plasma. Thirteen patients were analyzed. Patients who were alive at data cut-off, 19 January 2018, were censored at that date.

### Clinical characteristics

All 13 patients included in the analysis, were Caucasian, with a median age of 59 years, and had adenocarcinoma histology. The type of TKI-sensitizing *EGFR* mutation was a deletion in exon 19 in all the cases except 1 patient who harbored an uncommon *EGFR* mutation, G719A in exon 18. Sixty-nine percent of the patients had stage IV at diagnosis. The mean number of metastatic (M1) sites was 2 (range 1–5), with the lung, bone, and brain being the most frequent M1 sites (Table 1).

**Table 1:** Clinical and demographic characteristics of 13 patients treated with osimertinib

	N=13 n (number of patients)	%
Gender		
Male	3	23.1
Female	10	76.9
Ethnicity		
Caucasian	13	100
Age, years		
Mean (y)	61	
Median (y)	59	
Range (y)	37–70	
Histology		
Adenocarcinoma	13	100
Smoking history		
Never smoker	10	76.9
Ever smoker	3	23.1
Initial type of *EGFR* mutation		
del 19	12	92.3
G719A	1	7.7
Stage at diagnosis		
IA	1	7.7
IIIA	3^a^	23.1
IV	9	69.2
Baseline metastatic sites		
Mean number of M1 sites	2	
Range	1–5	
Baseline M1 sites		
Lung	9	69.2
Bone	6	46.1
Brain	3	23.1
Lymph nodes	2	15.4
Liver	1	7.7
Adrenal gland	1	7.7
Pleura	1	7.7

^a^: all patients received CT and PORT.

Abbreviations: CT, chemotherapy; del, deletion; M1, metastatic; PORT, postoperative radiotherapy; y, years.

All the patients had previously received a TKI (gefitinib in 46.1% of patients, afatinib in 15.4% of patients, and erlotinib in 38.5% of patients). The majority of patients (76.9%) received an EGFR TKI as first-line therapy, with best responses of a PR in 60% of patients and SD in 30% of patients (Table 2).

**Table 2:** Therapeutic history of 13 patients prior to osimertinib treatment

	N=13 n (number of patients)	%
**Prior treatments**		
Mean (number of treatments)	1.6	
Range	1–6	
Previous TKI	13	100
Prior CT	5	38.5^a, b^
**1st line TKI**		
N	10	76.9
Time to treatment failure (m)	11.25	
Range (m)	1–19	
Best response		
PR	6	60
SD	3	30
PD	1^c^	10
**2nd line TKI^d^**		
N	3	23.1
Time to treatment failure (m)	29	
Range (m)	5–76	
Best response		
PR	1	33.3
SD	2	66.6
**3rd line TKI**		
N	1	7.7
Time to treatment failure (m)	29	
Best response, PR	1	100
Type of TKI		
Gefitinib^e^	7	53.8
Afatinib	2	15.4
Erlotinib	5	38.5

^a^: This CT regimen included CT for advanced disease.

^b^: 2 patient received two different CT schedules.

^c^: This patient presented CNS PD, was treated with WBRT, and continued on TKI with TTF of 5 extra months.

^d^: Includes 1 patient that was reinduced with TKI.

^e^: 1 patients was reinduced with gefitinib after erlotinib.

Abbreviations: CNS, central nervous system; CT, chemotherapy; PD, progressive disease; PR, partial response; SD, stable disease; TKI, tyrosine kinase inhibitor; TTF, time to treatment failure; WBRT, whole brain radiotherapy.

In addition to T790M evaluation in serum/plasma, 6 patients underwent a rebiopsy after the TKI progression. T790M mutation was detected in tissue in 3 of these patients and was not detected in 2 patients, while in 1 patient the rebiopsy tissue was insufficient for the molecular analysis. The most frequent M1 sites prior to osimertinib treatment were the lung and the bone (61.5% and 30.8% of patients, respectively). Most (77%) patients presented Eastern Cooperative Oncology Group performance status (ECOG PS) 0-1 before starting osimertinib (Table 3).

**Table 3:** Specific details of the 13 patients prior to osimertinib therapy

	N=13 n (number of patients)	%
**T790M in serum**		
	13	100
**Tumor rebiopsy**		
no	7	58.8
yes	6	46.1
Presence of T790M in tissue	3^a^	50
M1 sites prior osimertinib initiation		
Mean (number of sites)	2.8	
Range	1–6	
Progressive M1 sites		
Lung	8	61.5
Bone	4	30.8
Brain	1	7.7
Pleura	1	7.7
Meningeal	1	7.7
Liver	1	7.7
ECOG PS prior to osimertinib initiation		
0	5	38.5
1	5	38.5
2	3	23.1

^a^: the rebiopsy material was insufficient for molecular testing in 1 patient and T790M was negative is tissue in 2 patients.

Abbreviations: ECOG, Eastern Cooperative Oncology Group; M1, metastatic; PS, performance status.

### Efficacy

Median patient follow-up time was 45.3 months (range 11.2–113.9). Median time on osimertinib therapy was 10.6 months (range 1.5–22.5). All 13 patients were evaluable for response to osimertinib. Six patients presented a partial response (PR) as best response (46.1%), while 4 patients presented SD (30.8%), for an overall disease control rate of 77%. In 3 out of the 4 patients with SD (75%), size of tumor lesions according to RECIST decreased in the first radiographic evaluation, while in 1 patient RECIST increased in 11%, resulting in a PD in the subsequent evaluation. Three patients presented PD in the first evaluation (23.1%). Two of these patients discontinued treatment with osimertinib due to radiological and clinical criteria, while in the other patient, osimertinib was maintained despite the RECIST progression due to clinical benefit.

Median PFS was 13.6 months (95% CI1.1-26.2). Median OS was 80.5 months (95% CI 12.8–148.2).

### Symptom burden and radiographic response

**Figure 1:**
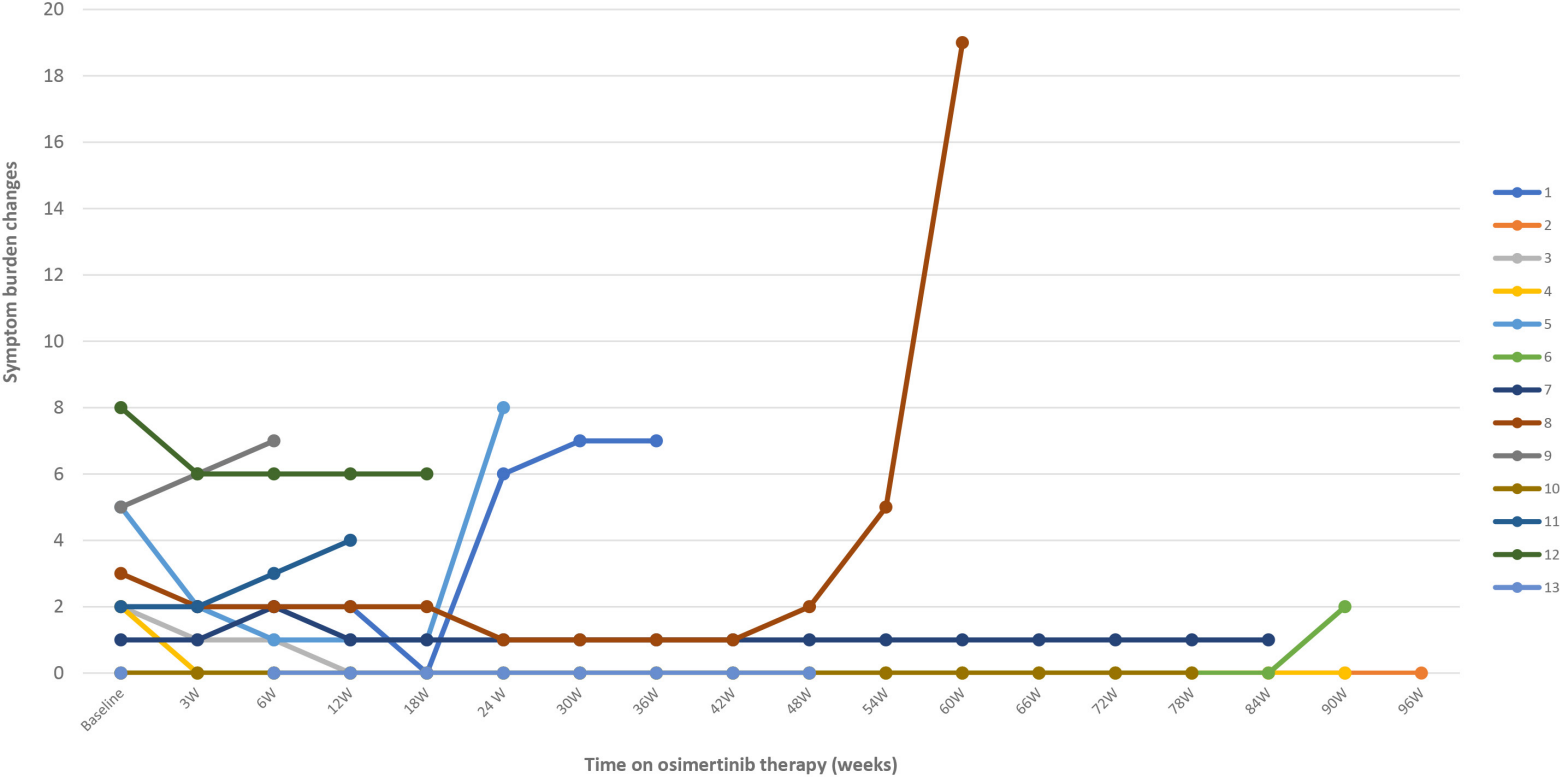
Changes in the symptomatic burden of the 13 patients with lung cancer with *EGFR*-mutations during treatment with osimertinib after acquired resistance to an EGFR TKI due to an *EGFR* T790M mutation W, weeks of therapy. Patient 1 presented symptomatic worsening in the context of an osteoporotic fracture. Patient 2 experienced clinical respiratory deterioration due to pulmonary embolism. Progressive disease and osimertinib-related pneumonitis were ruled out. Patient 6 experienced respiratory deterioration at week 90. Osimertinib-related pneumonitis was considered the most likely diagnosis at data cut-off. Patient 8 was admitted due to pneumonia with metachronous pleural effusion with severe respiratory deterioration that led to patient death.

**Figure 2:**
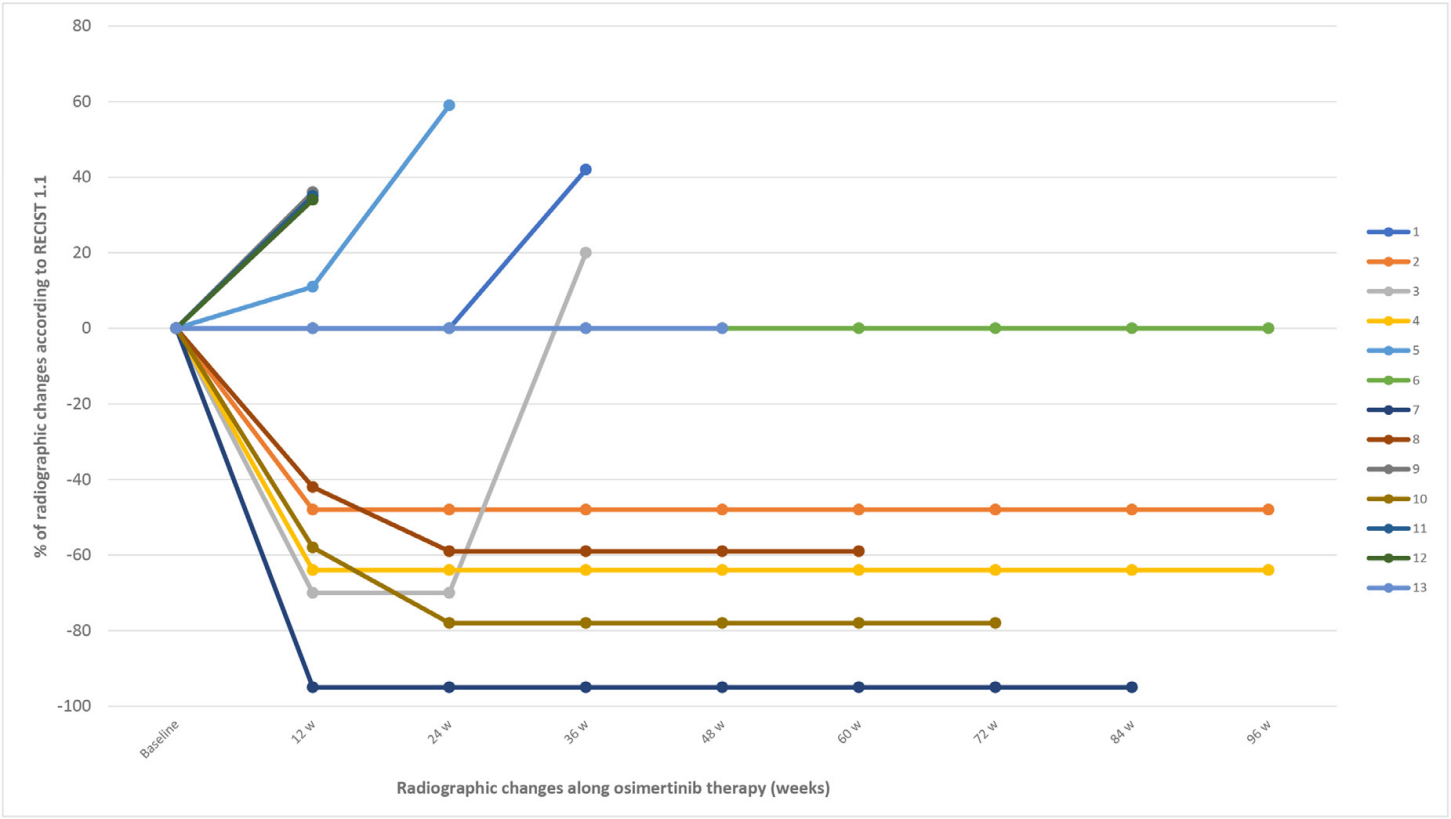
Radiographic changes according to RECIST 1.1 During treatment with osimertinib in the 13 patients with lung cancer with *EGFR*-mutations after acquired resistance to an EGFR TKI due to an *EGFR* T790M mutation. W, weeks of therapy.

The majority of patients included in this study presented few disease-related symptoms at baseline, with a median of symptomatic burden of 2 points (range 0–8) according to the proposed symptomatic scale. (Table 4) Four patients scored 0 at baseline. Nevertheless, all the patients were included in order to evaluate potential symptomatic changes during treatment. Four of the 6 patients (66.6%) who responded to osimertinib improved symptoms in the first visit after osimertinib initiation, while the other 2 patients remained symptomatically stable throughout treatment. One patient experienced transient respiratory deterioration in the context of a pulmonary embolism and disease progression was ruled out. Among patients with SD (n=4) as best response to osimertinib, 3 patients remained with stable symptoms (75%), while 1 patient presented a clinical improvement. Among patients with PD (n=3) during osimertinib treatment, 2 patients experienced clinical deterioration (66.6%) and osimertinib was stopped, while 1 patient experience symptomatic improvement and osimertinib was maintained despite radiographic progression according to RECIST criteria (Figures 1 and 2).

**Table 4:** Patients’ symptom burden scale based on the main symptoms related to lung cancer

Symptom/grade	0 or none	1 or mild	2 or moderate	3 or severe		
Cough						
Pain						
Weight loss						
Fatigue						
**Symptom/grade**	**0 or none**	**1 or mild**	**2 or moderate**	**3 or severe**	**4 or life-threatening**	**5 or death**
Anorexia						
Dyspnea						
Hemoptysis						

Based on the CTCAE 4.03 criteria, symptom changes were evaluated by using the information annotated in the medical records. Baseline and subsequent symptom burden was calculated by adding the overall score at each scheduled visit regarding the items included in the table.

### T790M in serum/plasma

**Figure 3:**
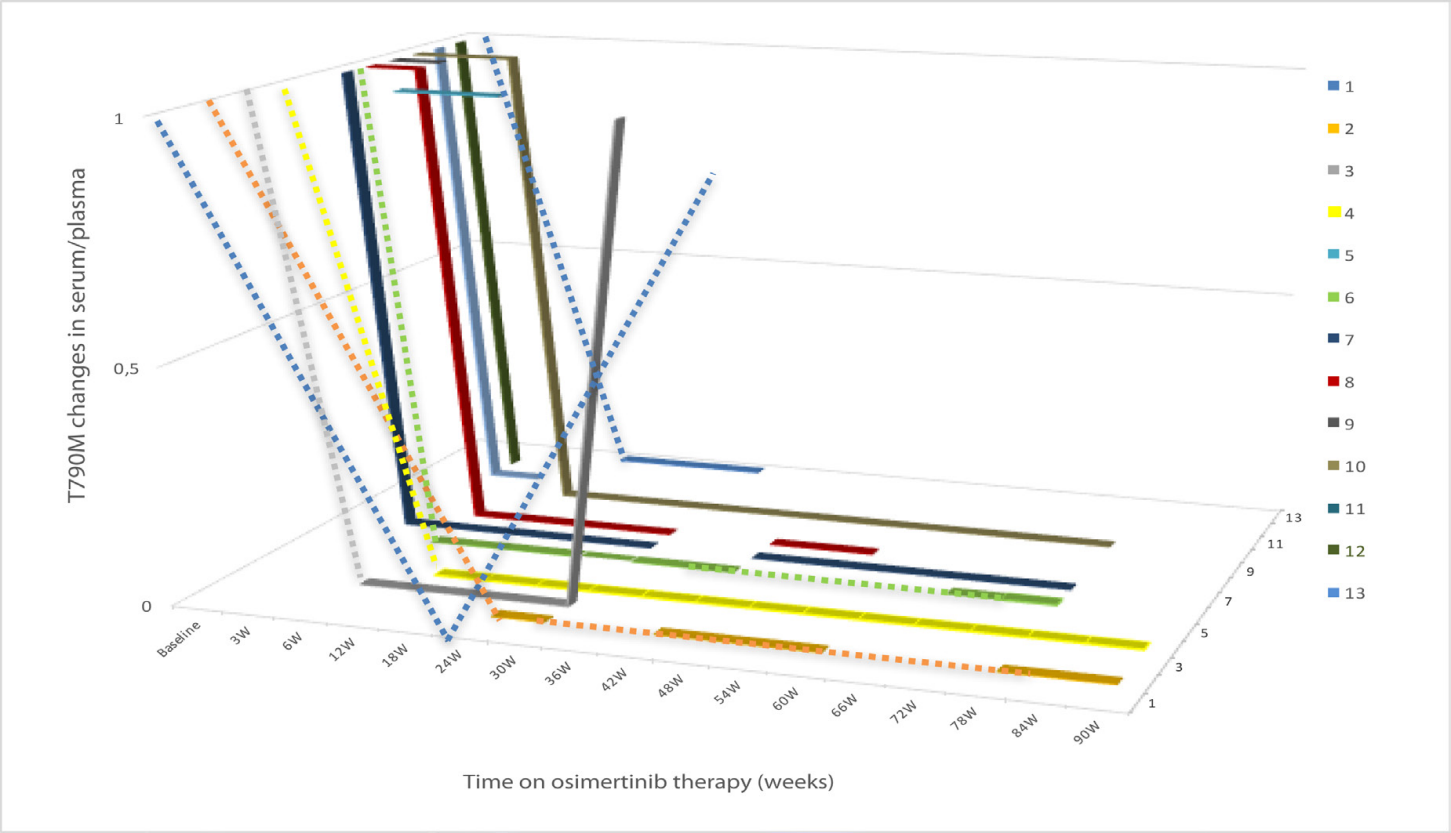
T790M evolution in plasma/serum at baseline and during treatment with osimertinib in the 13 patients with lung cancer with *EGFR*-mutations after acquired resistance to an EGFR TKI due to an *EGFR* T790M mutation W, weeks of therapy. **Each line corresponds to one individual patient**. The dashed sections correspond to periods of time where the plasma/serum sample was not drawn or no result was available.

The majority of patients had blood draws every 6 weeks according to the routine outpatient visits; however, 5 patients had blood draws as soon as 3 weeks after starting treatment with osimertinib. On the other hand, the first blood draw occurred in 3 patients at week 12, 21, and 24 weeks, respectively after osimertinib initiation. Some samples were missing, mainly due to clinical or technical reasons (Figure 3).

Overall, we identified three patterns of T790M behavior in serum/plasma during treatment with osimertinib. In one group of patients (n=9; 69.2%), T790M became undetectable in blood during osimertinib treatment; in some of the patients this occurred as early as 3 weeks after therapy initiation (n=3). All of the patients included in this group achieved a PR or SD (66.7% and 33.3%, respectively). Three out of the 9 patients (33.3%) included in this group reported symptom improvement, while the other 6 (66.7%) remained symptomatic stable; although the majority of these patients were paucisymptomatic at baseline (88.9%). Only 1 out of 9 patients (11.1%) presented PD after 6 months on osimertinib. In this patient, T790M became detectable again in the blood sample drawn at PD. As of the cut-off date, 2 patients in this subgroup had died: in 1 patient, the death was not considered related to disease progression but to an empyema, and the second patient developed a fatal pulmonary infection with metachronous pleural effusion. The severe condition of this patient prevented from performing a thoracentesis to rule out PD in the pleura. In both cases, T790M was negative in serum/plasma in the last sample drawn before death. Median PFS for the patients included on this group was no calculable (NC).

The second group of patients included two patients (15.4%) in whom T790M persisted during osimertinib treatment throughout the serial blood analyses. These patients presented a short-lasting SD and PD as best radiographic response. Median PFS for these patients was 1.8 months (95% CI, NC-NC). The symptomatic burden remained unchanged for 1 patient while the other presented a symptomatic improvement.

The third group included two patients (15.4%) who presented PD as best response to osimertinib even though they experienced a rapid disappearance of T790M in serum/plasma. Median PFS for these patients was 1.5 months (95% CI, NC-NC). One of the patients presented clinical benefit to osimertinib and the treatment was continued post-progression. Interestingly, the second patient presented PD as best response with symptomatic deterioration that led to osimertinib discontinuation. However, the rebiopsy of the progressive disease performed on the liver metastasis prior to osimertinib initiation did not reveal the presence of T790M. Unfortunately, shortly after disease progression was confirmed the patient suffered a stroke with disabling sequels that did not allow for subsequent rebiopsy or treatment.

## DISCUSSION

In the present study, we report the results of the prospective evaluation of detecting T790M in serum/plasma and its correlation with both clinical and radiographic response in patients with lung adenocarcinoma harboring *EGFR* mutations who were resistant to first- and second-generation TKIs and who were receiving treatment with osimertinib.

Several mechanisms of resistance to first- an second-generation TKIs have been described, with the acquisition of a T790M secondary mutation as the main mechanism of AR [21]. After the approval of osimertinib for *EGFR*-mutant patients with such a resistant mutation, its detection becomes of particular interest [22].

Using liquid biopsy as a technique to detect tumor abnormalities is of great interest, especially in the lung cancer field where the rebiopsy process can be challenging due to small sample size or lack of available tumor tissue for molecular assessment, which ranges from 15 to 23% according to different clinical series, or even the location or size of relapsing disease that may difficult the access for a new biopsy [23–26]. Moreover, tumor heterogeneity is a well-recognized issue that makes a single metastatic site not representative of the entire disease in a specific patient [21, 27–30]. In this regard, information obtained from blood could better depict this tumor heterogeneity and evolution of multiple metastatic sites during therapy [31]. Indeed, T790M has been reported exclusively in plasma in 31% and 35% of patients whose biopsy was T790M-negative or indeterminate [19, 24]. Additional advantages of the liquid biopsy include the potential use of a marker in a dynamic fashion to monitor the efficacy of the drug and the early detection of a resistant mutation. Moreover, the minimally invasive procedure required makes it of special interest for patients.

All of the patients in our study were included on the basis of a positive T790M liquid biopsy result. Rebiopsy could be performed only in 6 patients (46.1%) with a confirmative result in 3 of them. According to our series, 8 patients (38%) who ultimately benefited from osimertinib therapy were excluded from the present analysis since T790M was not detected in serum, strengthening the need of a rebiopsy for those patients whose result in liquid biopsy is negative. Moreover, results in serum/plasma resulted accurate for therapeutic decisions in 11 out of 13 patients (85%), regardless of the tissue results, which implies that biopsies could be obviated when T790M is detected in liquid biopsy such has been proposed [19].

Several studies have demonstrated that liquid biopsy can be useful to monitor the disappearance of a sensitizing *EGFR* mutations with EGFR TKI therapy, its reappearance after a period of treatment and the emergence of the T790M at progression [31, 32]. However, little has been reported regarding the behavior of T790M in liquid biopsy specifically during treatment with osimertinib. Some data in this regard were reported in both serum and urine samples in patients treated with rociletinib, a third generation TKI no longer available. In a small group of patients with serial urine samples, T790M levels decreased after a short period of therapy [20]. However, no longitudinal changes in plasma were reported [33]. Recently, a great spectrum of mechanisms of AR to osimertinib have been reported. Again, data on longitudinal changes along treatment with osimertinib have not been included [34].

In our study, blood samples were drawn periodically. According to our results, T790M monitoring during osimertinib therapy might predict those patients who will or will not respond to this drug. However, a solid conclusion cannot be drawn in terms of prediction of recurrence according to the reemergence of T70M in serum. Longer follow-up would be necessary to confirm if T790M mutation reappears in serum once the progression occurs, since a high proportion of patients are still on treatment with no evidence of progressive disease. Moreover, we can hypothesize that a proportion of patients will present the T790M once progression occurs, while other patients will acquire other mechanisms of resistance. T790M serial monitoring could be less useful for the subset of patients who develop additional mechanism of resistance to third-generation TKIs, including other novel mutations such as C797S, small cell lung cancer transformation, or MET amplification [34–36]. Our results are in contrast to other experiences that have reported contradictory results; patients with T790M loss in a cohort of 22 patients with AR to osimertinib experienced a shorter time to treatment failure than those who maintained T790M at resistance, suggesting the subclonal nature of this T790M in the blood and the potential emergence of a variety of alternative mechanisms of resistance [37].

Regarding the patient with a positive T790M in blood but negative in tissue, rapid decrease of T790M despite progression could be explained by T790M being present as a minor clone with the potential coexistence with other mechanisms of AR. Previously, patients with such molecular pattern have demonstrated a lower benefit from osimertinib compared with those who are plasma and tissue positive or plasma negative and tissue positive for T790M (19).

General efficacy results in our cohort of patients demonstrated a lower RR to osimertinib than reported in prior studies [12, 38]. Our series included a more pretreated population and patients with PS 2. On the other hand, OS in our cohort of patients is longer than previously reported. Several factors could potentially explain this result, such as the initial early stage diagnosis (30%), the presence of del19 (92.3%), the mean number of 1.6 metastatic sites and a high proportion of exclusive lung disease (46.1%). In addition, one patient has presented an outstanding survival that has lasted twelve years so far. The survival of this particular patient could have potentially influenced the OS of this small cohort of patients.

Different plasma genotyping assays have been used to isolate cfDNA [16, 18, 39, 40]. Each assay has advantages and drawbacks that should be considered. General limitations include the cost, the turn-around time, the imperfect accuracy, and the clinical context in which these tests are used. Ideally, plasma genotyping assays should be prospectively validated on paired plasma and tissue samples [32]. However, retrospective validation using plasma sample from large clinical trials paired with tissue sample have also been considered acceptable [18, 20]. A consistent trend among these well-validated plasma genotyping assays is the high specificity and a positive predictive value with a more limited sensitivity [16]. The advantages of the PNA-based PCR used in our study were its availability, its cost, and its favorable turn-around time (median of 3 days).

Our study includes several limitations. First, the number of patients included is small, but it actually reflects clinical practice and the target population being not possible to obtain robust conclusion and it should be considered an exploratory study. Second, the lack of assessment of other non-T790M mediated mechanisms of AR to osimertinib once the persistence or reemergence of T790M has been ruled out. Third, even after a long follow-up period, half of the patients were still on treatment and free of progression, and the molecular results in serum at progression are still unknown.

In conclusion, although our findings must clearly be interpreted with caution due to our small sample size, we have found that the disappearance or persistence of T790M in the serum/plasma in patients with lung adenocarcinoma and *EGFR* mutations and AR to first- and second-generation TKIs mediated by T790M may be a useful marker of symptomatic and radiographic evolution to osimertinib, a third-generation TKI able to target both *EGFR* sensitizing and T790M mutations. Longer follow-up is needed to establish if the subsequent emergence of T790M could be a marker of resistance, at least in the proportion of patients who did not develop additional mechanism of resistance to osimertinib.

## MATERIALS AND METHODS

### Patients

Patients with lung cancer with *EGFR* mutations who had progressed to a previous TKI and were selected for T790M inhibitor therapy based on positive T790M results in serum/plasma plus tissue were included in the analysis. The objectives were first, to describe the patient clinical characteristics by reviewing their medical records; second, to describe the tumor molecular characteristics; third, to detail the patients’ symptomatic burden at baseline; fourth, to specify patient clinical outcomes by evaluating symptom changes during osimertinib treatment using the Common Terminology Criteria for Adverse Events (CTCAE) 4.03 criteria; fifth, to interpret radiographic outcomes using the Response Evaluation Criteria In Solid Tumors (RECIST) 1.1 criteria during treatment; and seventh, to describe the changes in T790M mutation in serum/plasma during osimertinib treatment and try to correlate this changes with clinical and radiographic response.

All the patients received osimertinib as third-generation TKI at a standard dose of 80 mg/day orally as part of a clinical trial (NCT02474355) or per clinical practice. Treatment was maintained until disease progression or loss of clinical benefit. Visits were scheduled every six weeks. All the patients were followed up until death, withdrawal of consent, or loss of follow-up. Baseline is defined as the patient situation prior to osimertinib initiation.

In order to describe changes in the patients’ symptom burden and in the absence of formal questionnaires of quality of life, a scale that includes the main symptoms related to lung cancer was used. This scale was designed by reviewing the clinical records and assigning points to the following symptoms: cough, pain, dyspnea, hemoptysis, fatigue, anorexia, and weight loss (Table 4).

RECIST 1.1 was used to evaluate the radiographic response during treatment. CT scans were performed every 12 weeks, unless any clinical condition required additional imaging test to be performed. Response was evaluated by investigator.

### Molecular analyses

To evaluate T790M in tumor tissue, tumor genomic DNA was obtained from formalin-fixed paraffin-embedded tissue sections from the rebiopsy tumor sample. Briefly, samples were reviewed by a pathologist to estimate the percentage of neoplastic cells. Five μm unstained tissue sections were obtained, and manually macrodissected if necessary, to ensure more than 50% of neoplastic cell content. DNA extraction was performed with the Cobas^®^ DNA sample preparation kit (Roche Molecular Systems, Inc, Branchburg, NJ, USA) according to the manufacturer’s protocol. The extracted DNA was quantified using a DS-11 spectrophotometer (DeNovix Inc., Wilmington, DE, USA). *EGFR* mutations were determined with the Cobas^®^ EGFR Mutation Test v2 (Roche Molecular Systems, Inc, Branchburg, NJ, USA) according to the manufacturer’s recommendations. These analyses were performed at the Pathology Department of Hospital Universitari Germans Trias i Pujol, Badalona, Spain.

T790M mutations was tested in all patients in both serum and plasma as per our protocol. For T790M assessment in serum/plasma, peripheral blood was collected from patients in both a 10 ml Vacutainer tube for serum and a 10 ml EDTA tube for plasma (Becton Dickinson, Plymouth, UK). Tubes were centrifuged twice at 2300 rpm for 10 min and the supernatant transferred to sterile 1.5 ml tubes and stored at -20°C until use. Circulating DNA was purified, using the QIAamp DNA Blood Mini Kit (Qiagen, Hilden, Germany), according to the manufacturer’s instructions. This process was performed in independent duplicates. T790M mutations were examined by allelic discrimination Taqman assay performed in presence of a peptide-nucleic acid (PNA) designed to completely inhibit the amplification of the wt allele. All circulating-free DNA (cfDNA) samples were also tested without PNA to confirm the presence of DNA.

Primers, probes and PNA were described previously by our group [18, 41]. Blood draws were performed before starting osimertinib and periodically during the treatment. Blood draws were schedule every 6 weeks according to the clinical visits. These analyses were performed at the Molecular Biology Laboratory (Dr Rosell) at the Institute Germans Trias i Pujol (IGTP), Badalona, Spain.

All the patients signed the informed consent for their participation in the study, which included the clinical chart review, the molecular testing in the rebiopsy, when available, and periodic blood drawn and serial molecular testing. Approval was obtained from the IRB of our institution (RegTumTor2014).

### Statistical analyses

Data were described in terms of ranges, medians, frequencies and percentages. Overall survival (OS) was defined as the time from the initial diagnosis to death from any cause. PFS was defined as the time from starting osimertinib to documented disease progression or death. Time on treatment (ToT) was calculated from the stating date to the last dose of osimertinib. Patients who were still alive at the date of last contact were censored. OS and PFS were calculated with the Kaplan-Meier method. All statistical calculations were done with Microsoft Excel 2007 (Microsoft Corporation, NY, USA) and Statistical Package for the Social Sciences (SPSS Inc., Chicago, IL, USA) version 24 for Microsoft Windows.

## References

[1] Chen Y, Yang J, Li X, Hao D, Wu X, Yang Y, He C, Wang W, Wang J (2016). First-line epidermal growth factor receptor (EGFR)-tyrosine kinase inhibitor alone or with whole-brain radiotherapy for brain metastases in patients with EGFR-mutated lung adenocarcinoma. Cancer Sci.

[2] Pao W, Miller VA, Politi KA, Riely GJ, Somwar R, Zakowski MF, Kris MG, Varmus H (2005). Acquired resistance of lung adenocarcinomas to gefitinib or erlotinib is associated with a second mutation in the EGFR kinase domain. PLoS Med.

[3] Inukai M, Toyooka S, Ito S, Asano H, Ichihara S, Soh J, Suehisa H, Ouchida M, Aoe K, Aoe M, Kiura K, Shimizu N, Date H (2006). Presence of epidermal growth factor receptor gene T790M mutation as a minor clone in non-small cell lung cancer. Cancer Res.

[4] Price KA, Azzoli CG, Krug LM, Pietanza MC, Rizvi NA, Pao W, Kris MG, Riely GJ, Heelan RT, Arcila ME, Miller VA (2010). Phase II trial of gefitinib and everolimus in advanced non-small cell lung cancer. J Thorac Oncol.

[5] Johnson ML, Riely GJ, Rizvi NA, Azzoli CG, Kris MG, Sima CS, Ginsberg MS, Pao W, Miller VA (2011). Phase II trial of dasatinib for patients with acquired resistance to treatment with the epidermal growth factor receptor tyrosine kinase inhibitors erlotinib or gefitinib. J Thorac Oncol.

[6] Scagliotti GV, Krzakowski M, Szczesna A, Strausz J, Makhson A, Reck M, Wierzbicki RF, Albert I, Thomas M, Miziara JE, Papai ZS, Karaseva N, Thongprasert S (2012). Sunitinib plus erlotinib versus placebo plus erlotinib in patients with previously treated advanced non-small-cell lung cancer: a phase III trial. J Clin Oncol.

[7] Janjigian YY, Smit EF, Groen HJ, Horn L, Gettinger S, Camidge DR, Riely GJ, Wang B, Fu Y, Chand VK, Miller VA, Pao W (2014). Dual inhibition of EGFR with afatinib and cetuximab in kinase inhibitor-resistant EGFR-mutant lung cancer with and without T790M mutations. Cancer Discov.

[8] Reguart N, Rosell R, Cardenal F, Cardona AF, Isla D, Palmero R, Moran T, Rolfo C, Pallarès MC, Insa A, Carcereny E, Majem M, De Castro J (2014). Phase I/II trial of vorinostat (SAHA) and erlotinib for non-small cell lung cancer (NSCLC) patients with epidermal growth factor receptor (EGFR) mutations after erlotinib progression. Lung Cancer.

[9] Moran T, Felip E, Keedy V, Borghaei H, Shepherd FA, Insa A, Brown H, Fitzgerald T, Sathyanarayanan S, Reilly JF, Mauro D, Hsu K, Yan L, Johnson DH (2014). Activity of dalotuzumab, a selective anti-IGF1R antibody, in combination with erlotinib in unselected patients with Non-small-cell lung cancer: A phase I/II randomized trial. Exp Hematol Oncol.

[10] Moran T, Palmero R, Provencio M, Insa A, Majem M, Reguart N, Bosch-Barrera J, Isla D, Costa EC, Lee C, Puig M, Kraemer S, Schnell D, Rosell R (2017). A phase Ib trial of continuous once-daily oral afatinib plus sirolimus in patients with epidermal growth factor receptor mutation-positive non-small cell lung cancer and/or disease progression following prior erlotinib or gefitinib. Lung Cancer.

[11] Sequist LV, Soria JC, Goldman JW, Wakelee HA, Gadgeel SM, Varga A, Papadimitrakopoulou V, Solomon BJ, Oxnard GR, Dziadziuszko R, Aisner DL, Doebele RC, Galasso C (2015). Rociletinib in *EGFR*-mutated non-small-cell lung cancer. N Engl J Med.

[12] Jänne PA, Yang JC, Kim DW, Planchard D, Ohe Y, Ramalingam SS, Ahn MJ, Kim SW, Su WC, Horn L, Haggstrom D, Felip E, Kim JH (2015). AZD9291 in EGFR inhibitor-resistant non-small-cell lung cancer. N Engl J Med.

[13] Soria JC, Ohe Y, Vansteenkiste J, Reungwetwattana T, Chewaskulyong B, Lee KH, Dechaphunkul A, Imamura F, Nogami N, Kurata T, Okamoto I, Zhou C, Cho BC, FLAURA Investigators (2018). Osimertinib in Untreated *EGFR*-Mutated Advanced Non-Small-Cell Lung Cancer. N Engl J Med.

[14] Diehl F, Li M, Dressman D, He Y, Shen D, Szabo S, Diaz LA, Goodman SN, David KA, Juhl H, Kinzler KW, Vogelstein B (2005). Detection and quantification of mutations in the plasma of patients with colorectal tumors. Proc Natl Acad Sci U S A.

[15] Kimura H, Kasahara K, Kawaishi M, Kunitoh H, Tamura T, Holloway B, Nishio K (2006). Detection of epidermal growth factor receptor mutations in serum as a predictor of the response to gefitinib in patients with non-small-cell lung cancer. Clin Cancer Res.

[16] Sacher AG, Komatsubara KM, Oxnard GR (2017). Application of Plasma Genotyping Technologies in Non-Small Cell Lung Cancer: A Practical Review. J Thorac Oncol.

[17] Wu YL, Sequist LV, Hu CP, Feng J, Lu S, Huang Y, Li W, Hou M, Schuler M, Mok T, Yamamoto N, O’Byrne K, Hirsh V (2017). EGFR mutation detection in circulating cell-free DNA of lung adenocarcinoma patients: analysis of LUX-Lung 3 and 6. Br J Cancer.

[18] Karachaliou N, Mayo-de las Casas C, Queralt C, de Aguirre I, Melloni B, Cardenal F, Garcia-Gomez R, Massuti B, Sánchez JM, Porta R, Ponce-Aix S, Moran T, Carcereny E, Spanish Lung Cancer Group (2015). Association of EGFR L858R mutation in circulating free DNA with survival in the EURTAC trial. JAMA Oncol.

[19] Oxnard GR, Thress KS, Alden RS, Lawrance R, Paweletz CP, Cantarini M, Yang JC, Barrett JC, Jänne PA (2016). Association between plasma genotyping and outcomes of treatment with osimertinib (AZD9291) in advanced non-small-cell lung cancer. J Clin Oncol.

[20] Reckamp KL, Melnikova VO, Karlovich C, Sequist LV, Camidge DR, Wakelee H, Perol M, Oxnard GR, Kosco K, Croucher P, Samuelsz E, Vibat CR, Guerrero S (2016). A highly sensitive and quantitative test platform for detection of NSCLC EGFR mutations in urine and plasma. J Thorac Oncol.

[21] Sequist LV, Waltman BA, Dias-Santagata D, Digumarthy S, Turke AB, Fidias P, Bergethon K, Shaw AT, Gettinger S, Cosper AK, Akhavanfard S, Heist RS, Temel J (2011). Genotypic and histological evolution of lung cancers acquiring resistance to EGFR inhibitors. Sci Transl Med.

[22] Greig SL (2016). Osimertinib: First Global Approval. Drugs.

[23] Yu HA, Arcila ME, Rekhtman N, Sima CS, Zakowski MF, Pao W, Kris MG, Miller VA, Ladanyi M, Riely GJ (2013). Analysis of tumor specimens at the time of acquired resistance to EGFR-TKI therapy in 155 patients with EGFR-mutant lung cancers. Clin Cancer Res.

[24] Sundaresan TK, Sequist LV, Heymach JV, Riely GJ, Jänne PA, Koch WH, Sullivan JP, Fox DB, Maher R, Muzikansky A, Webb A, Tran HT, Giri U (2016). Detection of T790M, the acquired resistance EGFR mutation, by tumor biopsy versus noninvasive blood-based analyses. Clin Cancer Res.

[25] Douillard JY, Ostoros G, Cobo M, Ciuleanu T, Cole R, McWalter G, Walker J, Dearden S, Webster A, Milenkova T, McCormack R (2014). Gefitinib treatment in EGFR mutated caucasian NSCLC: circulating-free tumor DNA as a surrogate for determination of EGFR status. J Thorac Oncol.

[26] Bosc C, Ferretti GR, Cadranel J, Audigier-Valette C, Besse B, Barlesi F, Decroisette C, Lantuejoul S, Arbib F, Moro-Sibilot D (2015). Rebiopsy during disease progression in patients treated by TKI for oncogene-addicted NSCLC. Target Oncol.

[27] Piotrowska Z, Niederst MJ, Karlovich CA, Wakelee HA, Neal JW, Mino-Kenudson M, Fulton L, Hata AN, Lockerman EL, Kalsy A, Digumarthy S, Muzikansky A, Raponi M (2015). Heterogeneity underlies the emergence of EGFR^T790^wild-type clones following treatment of T790M-positive cancers with a third-generation EGFR inhibitor. Cancer Discov.

[28] Graziano P, de Marinis F, Gori B, Gasbarra R, Migliorino R, De Santis S, Pelosi G, Leone A (2015). *EGFR*-Driven Behavior and Intrapatient T790M Mutation Heterogeneity of Non-Small-Cell Carcinoma With Squamous Histology. J Clin Oncol.

[29] Kuiper JL, Heideman DA, Thunnissen E, Paul MA, van Wijk AW, Postmus PE, Smit EF (2014). Incidence of T790M mutation in (sequential) rebiopsies in EGFR-mutated NSCLC-patients. Lung Cancer.

[30] Hata A, Katakami N, Yoshioka H, Kaji R, Masago K, Fujita S, Imai Y, Nishiyama A, Ishida T, Nishimura Y, Yatabe Y (2015). Spatiotemporal T790M heterogeneity in individual patients with EGFR-mutant non-small-cell lung cancer after acquired resistance to EGFR-TKI. J Thorac Oncol.

[31] Yanagita M, Redig AJ, Paweletz CP, Dahlberg SE, O’Connell A, Feeney N, Taibi M, Boucher D, Oxnard GR, Johnson BE, Costa DB, Jackman DM, Jänne PA (2016). A prospective evaluation of circulating tumor cells and cell-free DNA in EGFR-mutant non-small cell lung cancer patients treated with erlotinib on a phase II trial. Clin Cancer Res.

[32] Sacher AG, Paweletz C, Dahlberg SE, Alden RS, O’Connell A, Feeney N, Mach SL, Jänne PA, Oxnard GR (2016). Prospective Validation of Rapid Plasma Genotyping for the Detection of *EGFR* and *KRAS* Mutations in Advanced Lung Cancer. JAMA Oncol.

[33] Karlovich C, Goldman JW, Sun JM, Mann E, Sequist LV, Konopa K, Wen W, Angenendt P, Horn L, Spigel D, Soria JC, Solomon B, Camidge DR (2016). Assessment of EGFR Mutation Status in Matched Plasma and Tumor Tissue of NSCLC Patients from a Phase I Study of Rociletinib (CO-1686). Clin Cancer Res.

[34] Lin CC, Shih JY, Yu CJ, Ho CC, Liao WY, Lee JH, Tsai TH, Su KY, Hsieh MS, Chang YL, Bai YY, Huang DD, Thress KS, Yang JC (2018). Outcomes in patients with non-small-cell lung cancer and acquired Thr790Met mutation treated with osimertinib: a genomic study. Lancet Respir Med.

[35] Kim TM, Song A, Kim DW, Kim S, Ahn YO, Keam B, Jeon YK, Lee SH, Chung DH, Heo DS (2015). Mechanisms of Acquired Resistance to AZD9291: A Mutation-Selective, Irreversible EGFR Inhibitor. J Thorac Oncol.

[36] Oxnard GR, Thress K, Paweletz C, Stetson D, Dougherty B, Lai Z, Markovets A, Felip E, Vivancos A, Kuang Y, Sholl L, Redig AJ, Cantarini M (2015). Mechanisms of Acquired Resistance to AZD9291 in EGFR T790M Positive Lung Cancer ORAL17.07. J Thorac Oncol.

[37] Oxnard G, Hu Y, Mileham K, Tracy P, Feeney N, Sholl L, Paweletz C, Thress K, Jänne P (2017). Osimertinib Resistance Mediated by Loss of EGFR T790M Is Associated with Early Resistance and Competing Resistance Mechanisms. J Thorac Oncol.

[38] Mok TS, Wu YL, Ahn MJ, Garassino MC, Kim HR, Ramalingam SS, Shepherd FA, He Y, Akamatsu H, Theelen WS, Lee CK, Sebastian M, Templeton A, AURA3 Investigators (2017). Osimertinib or Platinum-Pemetrexed in EGFR T790M-Positive Lung Cancer. N Engl J Med.

[39] Jahr S, Hentze H, Englisch S, Hardt D, Fackelmayer FO, Hesch RD, Knippers R (2001). DNA fragments in the blood plasma of cancer patients: quantitations and evidence for their origin from apoptotic and necrotic cells. Cancer Res.

[40] Stroun M, Lyautey J, Lederrey C, Olson-Sand A, Anker P (2001). About the possible origin and mechanism of circulating DNA apoptosis and active DNA release. Clin Chim Acta.

[41] Rosell R, Carcereny E, Gervais R, Vergnenegre A, Massuti B, Felip E, Palmero R, Garcia-Gomez R, Pallares C, Sanchez JM, Porta R, Cobo M, Garrido P, Spanish Lung Cancer Group in collaboration with Groupe Français de Pneumo-Cancérologie and Associazione Italiana Oncologia Toracica (2012). Erlotinib versus standard chemotherapy as first-line treatment for European patients with advanced EGFR mutation-positive non-small-cell lung cancer (EURTAC): a multicentre, open-label, randomised phase 3 trial. Lancet Oncol.

