# Correction: Monitoring *EGFR*-T790M mutation in serum/plasma for prediction of response to third-generation EGFR inhibitors in patients with lung cancer

**DOI:** 10.18632/oncotarget.26855

**Published:** 2019-04-02

**Authors:** Teresa Morán, Eudald Felip, Joaquim Bosch-Barrera, Itziar de Aguirre, Jose Luis Ramirez, Carles Mesia, Enric Carcereny, Diana Roa, Elia Sais, Yolanda García, Remei Blanco, Silvia Sanchez, Claudia Rosa Villacorta, Cristina Queralt, Jose María Velarde, Rafael Rosell

**Affiliations:** ^1^ Medical Oncology Department, Catalan Institute of Oncology - Badalona, Hospital Universitari Germans Trias i Pujol, Badalona, Department of Medicine, Universitat Autònoma de Barcelona (UAB), Barcelona, Spain; ^2^ Medical Oncology Department, Catalan Institute of Oncology-Girona, Hospital Universitari Doctor Trueta Girona, Girona, Spain; ^3^ Molecular Biology Laboratory of Cancer Dr. Rosell, Can Ruti Campus: Institute Germans Trias i Pujol (IGTP), Catalan Institute of Oncology, Badalona, Spain; ^4^ Medical Oncology Department, Catalan Institute of Oncology - Hospital Duran i Reynalds, l’Hospitalet de Llobregat, Barcelona, Spain; ^5^ Medical Oncology Department, Hospital Parc Taulí, Sabadell, Barcelona, Spain; ^6^ Medical Oncology Department, Consorci Sanitari de Terrassa, Terrassa, Barcelona, Spain; ^7^ Research Nurse Team, Catalan Institute of Oncology - Badalona, Hospital Universitari Germans Trias i Pujol, Badalona, Spain; ^8^ Statistics Department, Fundació Germans Trias i Pujol, Badalona, Spain; ^9^ Department of Medicine, Universitat Autònoma de Barcelona (UAB), Barcelona, Spain

**This article has been corrected:** The correct affiliation information is given below:

^1^ Medical Oncology Department, Catalan Institute of Oncology - Badalona, Hospital Universitari Germans Trias i Pujol, Badalona, Department of Medicine, Universitat Autònoma de Barcelona (UAB), Barcelona, Spain

Original article: Oncotarget. 2018; 9:27074-27086. https://doi.org/10.18632/oncotarget.25478

